# Adoption or Placement in Foster Care and Catch-up in Linear Growth and Development: A Meta-Analysis of Individual Participant Data

**DOI:** 10.1016/j.advnut.2025.100395

**Published:** 2025-02-22

**Authors:** Jef L Leroy, Moira Donahue Angel, Edward A Frongillo

**Affiliations:** 1 Nutrition, Diets, and Health Unit, International Food Policy Research Institute, Washington, DC, United States; 2 Department of Health Promotion, Education, and Behavior, Arnold School of Public Health, University of South Carolina, Columbia, SC, United States

**Keywords:** children, linear growth, stunting, adoption, catch-up growth, child development

## Abstract

The ability of children to recover from linear growth retardation, often referred to as catch-up growth, has intrigued researchers for many decades. Whether adoption from a low-income to a high-income setting, which provides a comprehensive improvement in the conditions that cause children to not grow well, leads to catch-up growth is unknown. We estimated the association of adoption (or placement in foster care) with catch-up in linear growth and child development before 5 y of age. We conducted a 2-stage meta-analysis using individual participant data for linear growth. We obtained study-specific and subgroup estimates and pooled the estimates using random-effects models. Sensitivity analyses were used to assess the robustness of our findings. A review of child-development outcomes was conducted. We included 485 children under 5 y of age from 9 adoption studies. At baseline, children had a mean age of 15.8 mo and a length deficit of 3.9 cm. Adoption reduced this gap by 77% or 3.0 cm (95% confidence interval [CI]: 1.9, 4.1 cm; mean age: 32.3 mo). Catch-up growth was found in both girls (3.6 cm; 95% CI: 2.9, 4.2 cm) and boys (2.5 cm; 95% CI: 1.9, 3.1 cm) and in children adopted after the age of 24 mo (2.2 cm; 95% CI: 0.6, 3.7 cm). The sensitivity analyses did not change any of the substantive findings. The magnitude of catch-up in child development (mean reduction in deficit of 46%) was smaller than that in linear growth. Catch-up in linear growth in children under 5 is biologically possible when the environment is improved profoundly and comprehensively. Partial reversal of the accumulated height deficit is more likely than recovery in developmental outcomes, which highlights the need to ensure all children grow and develop in environments that prevent deficits from occurring rather than trying to correct them.

This review was registered at PROSPERO as CRD42022298715 (https://www.crd.york.ac.uk/PROSPEROFILES/298715_PROTOCOL_20220429.pdf).

**Statement of significance**

This study, to our knowledge, the first meta-analysis of adoption using individual participant data, showed that adoption reduced children’s accumulated height deficit by ∼3 cm. Catch-up in linear growth was more likely than recovery in developmental outcomes, highlighting the need to ensure that children grow and develop in environments that prevent deficits from occurring rather than trying to correct them.

## Introduction

Children who live in environments that do not provide enough food or foods with enough nutrients, that cause them to get sick repeatedly, and that lack accessible high-quality health services often experience linear growth retardation. The prevalence of stunting, defined as children with a height-for-age *z*-score (HAZ) < −2 SD, is therefore used as a measure of the deficient environment to which a population of children has been exposed in the past or is currently exposed [1]. The ability of children to recover from linear growth retardation, often referred to as catch-up growth, has intrigued researchers for several decades.

Several observational studies have concluded that catch-up growth is possible [2,3] and that it should be promoted as a strategy to improve child development [2,4]. These studies, however, do not meet the criteria for catch-up growth as defined by Boersma and Wit [5]: a growth-inhibiting condition, which causes a reduction in linear growth velocity, is alleviated or compensated and followed by higher-than-normal velocity [6]. Many observational studies that have claimed demonstrating catch-up in linear growth [2,3] did not assess linear growth before and after the removal of a growth-inhibiting condition [2,3]. Furthermore, catch-up growth in height requires children to grow substantially faster than the expected linear growth velocity (for their age and sex) so they can make up for the lost growth in height [7]. Experimental studies of nutrition interventions implemented in low-income and middle-income countries have shown improved linear growth [8], but the size of the improvement is too small for catch-up growth to occur [7]. Catch-up growth in the absence of an intervention is thus unlikely. A second concern relates to the claim that linear growth and child development are causally linked; no evidence supports this claim [1].

The most comprehensive improvement in the conditions that cause children to not grow well is provided by adoption. Adoption from a resource-poor to a high-income setting leads to a large and sustained improvement in a child’s environment with respect to diet, water, sanitation, hygiene, and opportunities to receive responsive care in a stable household setting. Adoption studies thus provide evidence of the maximum attainable effect on linear growth when environmentally inhibiting conditions are alleviated.

A recent review of the literature showed that adoption from a low-income to high-income setting can lead to catch-up in linear growth [7]. A key limitation of the review, however, was the use of the reported mean values rather than individual-level data. In addition, many of the studies used a growth reference instead of the WHO growth standard; the review’s use of the WHO standard to derive absolute height values from the reported *z*-scores may have introduced some imprecision. The analysis presented in this study used individual participant data. Key advantages of analyses of individual data include the ability to check data quality and to conduct subgroup analyses. Our primary objective was to estimate the association of adoption (or placement in foster care) before 5 y of age with catch-up in linear growth and to assess whether child sex and age at adoption modified the association. Our secondary objective was to review the association of adoption with child development.

## Methods

The protocol for this meta-analysis was registered at PROSPERO as CRD42022298715. We followed the guidelines from PRISMA of Individual Patient Data [9].

### Search strategy and selection criteria

We conducted a 2-stage meta-analysis using individual participant data. We considered all studies identified for a recently published literature review [7]. For that review, the authors screened all studies included in and citing the 1994 seminal review on the reversibility of stunting by Martorell et al. [10] and the more recent review by van Ijzendoorn et al. [11] on plasticity of growth after international adoption. To identify recent (i.e., published after 2006) studies examining the link between adoption and linear growth and adoption, Leroy et al. [7] searched PubMed using the search string “(catch-up OR recovery OR growth OR height) AND (adopt OR orphan) AND (child OR infant).” To conduct the analyses in the review, children’s height in centimeters was calculated using child sex, mean age, and mean HAZ as reported in the articles. Consequently, only studies that provided information on the proportion of boys and girls and on children’s mean age could be included. Studies that did not provide this information were excluded from the published review but were considered for this study (Supplemental Table 1). We included studies that met the following criteria: *1*) adoption (or placement in foster care) of children living in low-income and middle-income countries (as defined by the World Bank for the year that the data were collected) or comparable disadvantaged conditions leading to growth retardation; *2*) adoption to (or placement in foster care in) a setting without resource constraints that limit linear growth; and *3*) studies with longitudinal follow-up data on individual children.

Authors of all studies meeting the eligibility criteria were contacted and invited to share their data sets. We requested missing variables or clarifications, as needed, from the study investigators. The investigators of 1 study [12] could not share individual data and were asked to produce individual-level estimates of the overall effect of adoption on growth retardation and of the effect stratified by the predefined effect modifiers within the trial population. We provided the data analytical plan, data dictionary, and R syntax to perform the analyses.

When reviewing the included studies (and after registering the study protocol), we found that several articles reported on changes in child development, an outcome we did not preregister. Since the comparison of catch-up in linear growth and child development following adoption could lead to important insights, we decided to include child development in the review. For each study meeting the inclusion criteria, we searched the literature for studies on the same cohort that assessed child development. We searched PubMed for the name of the study or cohort when available, names of key authors of the parent study, and names of the principal investigators as registered on clinicaltrials.gov. Searches that produced excessive (>10,000) results were narrowed down by the addition of search terms “(adoption) OR (adopt) OR (adoptees).” Titles and abstracts were screened for relevance. Inclusion was limited to studies that assessed development using the same measure on the same children before and after adoption and before 5 y of age.

### Study outcomes

The primary outcome was height-for-age difference (HAD), which is the difference between the measured height and the median sex-specific and age-specific height obtained from the WHO growth standard [13]. Catch-up growth was defined as an absolute linear growth velocity (e.g., centimeters per month) higher than the expected linear growth velocity for age and sex using the WHO growth standard [13]. This is mathematically equal to a reduction in the absolute height deficit or HAD [7]. We do not report on HAZ as primary outcome because it is inappropriate to evaluate changes in this measure as children age [14]. The calculation of HAZ makes it impossible to assess whether a change in HAZ with age is due to changes in the numerator (the magnitude of the deficit) or to changes in the denominator (the increasing SD with age) [14,15]. To allow readers to compare the size of changes in HAZ following adoption with the impact of nutrition interventions, we report HAZ estimates in Supplemental Material. We calculated HAZ using the WHO standard. The child development measures varied considerably across studies, making a meta-analysis of these measures infeasible.

### Data preparation

We checked for data completeness by comparing the sample sizes in the received data sets against the corresponding publications when this information was available. Child sex and age, dates of adoption, and dates of baseline and follow-up measurements were extracted from the data. Where studies provided age in whole months [[16], [17], [18], [19]], we added a half month to each age before deriving HADs from the WHO standard.

We only included children with child length or height collected within 3 mo before or after adoption (or foster care placement) and a second measurement collected ≥3 mo after adoption and before 60 mo of age. Before calculating HAD and HAZ, we subtracted 0.7 cm from recumbent length measurements of children over 24 mo of age and added 0.7 cm to standing height measurements of children under 24 mo of age, following WHO protocol. For 3 studies [16,17,19] in which the method of measurement (recumbent or standing) was not documented and could not be recalled by the principal investigator, we assumed that measurements followed standard WHO protocol: recumbent for children <24 mo of age and standing for children 24 mo and older. Identifying biologically implausible values for HAD was done indirectly through inspecting HAZ values for acceptable SDs and to be within published WHO acceptable ranges (HAZ <−6 or >6) [20]. Biologically implausible values were inspected for errors and removed from the analyses.

### Data analysis

We estimated the mean and SD of the child-level outcomes at baseline (i.e., the time of adoption or foster care placement) and follow-up for each study separately and for all children combined following the Cochrane Handbook guidance [21]. The effect of adoption (or placement in foster care) was assessed by comparing the child’s HAD before and after adoption. If studies had collected data at >2 times, we restricted the analyses to the baseline measurement closest to the time of adoption and the follow-up measurement corresponding to the highest age (but below 5 y of age) after adoption. We excluded children whose baseline measurements were taken >3 mo before or after arriving in their new environment. In cases where an exact age (or date) of arrival was not available and consequently could not be used to determine when baseline measurement occurred in relation to the child’s arrival, we used the eligibility criteria of the original study or the aggregate-level information reported in the publication or provided by the study team.

The meta-analysis used a 2-stage method. In the first stage, the association of adoption with linear growth was estimated using a mixed-effects model with child-specific random intercepts and a time dummy (before or after adoption) and child age and child sex as fixed effects. The estimates were pooled in the second-stage using an inverse-variance (DerSimonian and Laird) random-effects model. This reflects our assumption that the association of adoption with linear growth could vary from study to study. *I*^2^ statistics were used to assess heterogeneity [22]. First-stage estimates were conducted using Stata version 17.0 and R version 4.3.2. All second-stage estimates were conducted with the Stata programs ipdmetan and metan [23,24].

We explored the heterogeneity of the association by child sex and by age of adoption or placement into foster care using the same 2-stage method. Analyses by child sex were adjusted for child age; stratified analyses by child’s age at adoption or placement into foster care were adjusted for child age and sex. We used 3 age-of-adoption groups: before 12 mo, between 12 and 24 mo, and after 24 mo.

We conducted a series of sensitivity analyses to check the robustness of our findings. We implemented second-stage, fixed-effects analyses and compared the findings to the random-effects results. We also tested how sensitive the results were when restricting the analyses to observations that met the specifications for baseline measurements in the original protocol. Finally, we checked the sensitivity of results to risk of bias by limiting the analyses to the studies with low risk of bias.

The Bucharest Early Intervention Project (BEIP) was the only RCT [25] in our study. For consistency, only the intervention arm was considered in the primary analysis. In a separate analysis, we took advantage of the randomized intervention design. The BEIP study randomly assigned institutionalized children in Romania to either foster care (intervention arm) or to remain in institutional care known to be resource deprived (control arm). We used a mixed model with child as a random effect and treatment group, time, a treatment × time interaction, child age, and sex as fixed effects. Many of the children randomly assigned to institutional care eventually were placed in foster care or adopted. Thus, the intention-to-treat analysis produced conservative estimates of the true effect of placement into better environments.

Child development was assessed using a variety of measures, including the Denver Development Scales, Mullen Scales of Early Learning, and the Bayley Scales of Infant Development. The child development outcomes were thus too heterogeneous to conduct a meta-analysis. To the extent possible, we expressed the change as the percent reduction in the size of the development gap.

### Risk-of-bias assessment

We assessed risk of bias for linear growth in domains that we deemed applicable to the intervention studies available for this review. They were selected from the revised Cochrane risk-of-bias tool for randomized trials [26] and excluded domains that were relevant to randomized clinical trials only, such as nonrandom allocation sequence and nonblinding to treatment. We assessed risk of bias in 4 domains: bias due to deviations from intended interventions, bias due to missing outcome data, bias in measurement of the outcome, and bias in selection of the reported result. Risk of bias was summarized across all domains for each study. The following considerations guided our risk-of-bias assessment. First, the main objective of our study was to assess whether catch-up growth is biologically possible. Our primary concern in risk-of-bias assessment was thus to identify bias that would lead to false-positive results. Second, there is no good understanding of the determinants of the potential for catch-up growth. Missing baseline measurements were thus considered to pose a low risk of biasing our results. Missing follow-up measurements, however, were considered to pose at least some risk of bias.

### Deviation from the registered study protocol

We deviated from the registered study protocol in 3 ways. First, the original protocol defined a baseline period as a period of no >3 mo before or 1 mo after adoption or foster care placement. We widened this narrow interval and accepted measurements taken within 3 mo before or after adoption or foster care placement. The change was implemented for the following reasons: *1*) 1 study did not have any baseline values as originally defined but did have baseline measurements using the widened interval; and *2*) our sample size increased, which allowed us to better explore heterogeneity. We made this decision acknowledging that this change would likely produce more conservative estimates on catch-up growth as any growth occurring in the period between adoption and the baseline measurement would not be captured in the impact estimate. The extent to which these modifications changed the findings was assessed in sensitivity analyses. Second, we intended to explore heterogeneity by how long children were exposed to the improved environment, using a cutoff of 6 mo to define a short compared with long exposure, but only 1 study could be used for this comparison [27]. The analysis on the association with exposure length was thus dropped. Third, we added the review of the evidence on child development outcomes.

## Results

**FIGURE 1:**
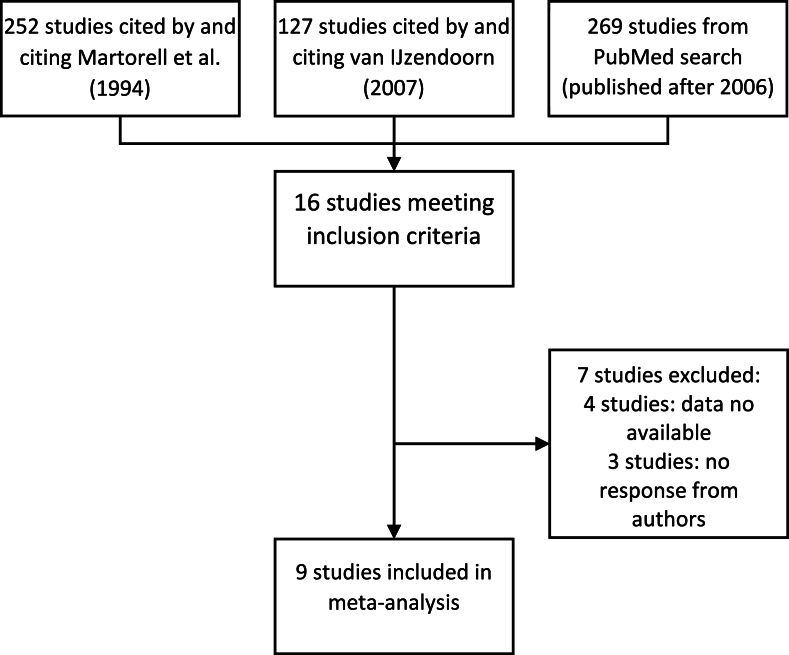
Study selection.

Eight adoption studies and 1 foster care study were included in the meta-analysis providing data on 485 children meeting the inclusion criteria (Figure 1, TABLE 1, TABLE 2) [12,18,19,25,[27], [28], [29], [30], [31]]. Children were adopted from Eastern Europe, Asia, Central and South America, and West and East Africa and moved to new home environments in the Netherlands, Italy, United States, United Kingdom, and Spain. The Romanian children in the foster care study were placed with foster families in their own country [25].

**TABLE 1:** Studies included in the meta-analysis.

Study	Countries of origin original study	Countries of origin of children included in meta-analysis	Setting before adoption	Countries of adoption
Rutter, 1998 [28]	Romania	Romania	Predominantly institutional care	United Kingdom
Miller et al., 2010 [27]	Russia, Kazakhstan, and Ukraine	Unknown (data on country of origin not shared by authors)	Institutional care	United States
van den Dries et al., 2010 [29]	China	China	Institutional and foster care	Netherlands
Palacios et al., 2011 [18]	China, Colombia, Guatemala, India, Romania, and the Russian Federation	China, Colombia, Guatemala, India, Romania, and the Russian Federation	Institutional and foster care	Spain
Park et al., 2011 [30]	Russia, China, and Ukraine (+8 other unnamed countries)	Unknown (data on country of origin not shared by authors)	Institutional care	United States
Fuglestad et al., 2016 [12]	Russia, Kazakhstan, Ethiopia, and China	Unknown (data on country of origin not shared by authors)	Institutional care	United States
Matthews et al., 2016 [31]	Albania, Chile, China, Colombia, Dominican Republic, Ecuador, Ethiopia, India, Korea, Lithuania, Mexico, Moldova, Paraguay, Peru, Romania, Russia, and other	China, Colombia, Dominican Republic, Romania, and Mexico	Information not provided	United States
Johnson et al., 2018 [25]	Romania	Romania	Institutional care	Romania
Canzi et al., 2021 [19]	Burkina Faso, Congo, Ethiopia, Tanzania, China, Thailand, Vietnam, Bolivia, Chile, and Russian Federation	Burkina Faso, Congo, Ethiopia, Tanzania, China, Vietnam, and Russian Federation	Institutional care	Italy

**TABLE 2:** Child age, adoption, and anthropometric characteristics by study and by subgroup.

Study	*N*	Male (%)	Age at adoption^1^ (mo)	Adoption exposure period (mo)	Baseline				Follow-up			
					Age^2^ (mo)	HAD (cm)	HAZ	Stunting (%)	Age^2^ (mo)	HAD (cm)	HAZ	Stunting (%)
Studies												
Rutter, 1998 [28]	74	47.3	7.7 ± 6.2	42.0 ± 7.1	7.6 ± 6.5	−4.5 ± 3.3	−1.9 ± 1.4	47.3	50.4 ± 1.5	−2.6 ± 3.8	−0.6 ± 0.9	4.1
Miller et al., 2010 [27]	125	52.8	19.2 ± 9.0	6.2 ± 0.6	19.7 ± 9.1	−4.5 ± 3.6	−1.5 ± 1.1	31.2	25.8 ± 9.1	−2.6 ± 3.7	−0.8 ± 1.1	9.6
van den Dries et al., 2010 [29]	85	0.0	13.0 ± 1.3	4.2 ± 0.6	15.3 ± 1.4	−2.3 ± 2.7	−0.8 ± 1.0	11.8	19.5 ± 1.5	−2.4 ± 3.2	−0.8 ± 1.1	14.1
Palacios et al., 2011 [18]	83	27.7	14.2 ± 7.8	27.3 ± 9.6	14.8 ± 7.8	−4.5 ± 5.6	−1.7 ± 2.0	42.2	42.1 ± 10.8	−1.5 ± 5.2	−0.4 ± 1.3	7.2
Park et al., 2011 [30]	11	90.9	19.0 ± 8.8	5.6 ± 2.6	19.5 ± 8.7	−4.9 ± 3.8	−1.7 ± 1.1	27.3	24.7 ± 9.1	−1.7 ± 5.6	−0.4 ± 1.8	18.2
Fuglestad et al., 2016 [12]	47	40.4	11.9 ± 2.5	6.1 ± 0.7	12.6 ± 2.5	−3.7 ± 3.6	−1.5 ± 1.4	36.2	18.7 ± 2.6	−2.3 ± 3.3	−0.8 ± 1.1	19.1
Matthews et al., 2016 [31]	5	20.0	13.2 ± 12.6	12.4 ± 12.7	13.9 ± 13.0	−5.0 ± 4.0	−1.8 ± 1.3	60.0	26.3 ± 15.5	−8.6 ± 5.4	−2.5 ± 1.2	40.0
Johnson et al., 2018 [25]	43	46.5	22.4 ± 7.5	19.4 ± 7.0	21.0 ± 7.4	−3.4 ± 2.9	−1.1 ± 0.9	16.3	41.7 ± 2.6	−1.1 ± 3.7	−0.3 ± 0.9	2.3
Canzi et al., 2021 [19]	12	50.0	26.2 ± 14.3	11.7 ± 2.9	26.7 ± 14.3	−4.7 ± 5.4	−1.4 ± 1.5	33.3	38.3 ± 13.7	−1.8 ± 6.5	−0.4 ± 1.6	16.7
All studies combined	485	37.1	15.2 ± 8.5	16.3 ± 14.7	15.8 ± 8.5	−3.9 ± 3.9	−1.4 ± 1.4	31.5	32.3 ± 13.6	−2.2 ± 4.1	−0.6 ± 1.2	10.1
Subgroup^3^												
Child sex												
Male	169	100.0	16.5 ± 10.0	17.8 ± 15.1	16.8 ± 10.0	−4.3 ± 3.9	−1.6 ± 1.4	37.2	34.9 ± 13.6	−2.2 ± 3.6	−0.6 ± 1.0	16
Female	215	0.0	14.8 ± 8.6	20.5 ± 15.1	15.0 ± 8.7	−4.2 ± 4.3	−1.6 ± 1.5	34.4	35.8 ± 13.4	−2.1 ± 4.6	−0.5 ± 1.2	10.7
Child age at adoption^1^ (mo)												
<12.0	65	33.8	8.8 ± 2.3	20.9 ± 13.9	9.1 ± 2.4	−2.8 ± 4.1	−1.2 ± 1.8	27.7	30.1 ± 12.9	−0.2 ± 5.0	−0.1 ± 1.4	4.6
12.0–23.9	127	44.1	16.7 ± 3.4	14.1 ± 10.7	17.1 ± 3.3	−4.3 ± 3.9	−1.6 ± 1.4	33.1	31.4 ± 11.2	−1.9 ± 3.1	−0.6 ± 0.9	5.5
≥24	59	52.5	31.2 ± 5.5	12.1 ± 7.1	31.1 ± 5.9	−5.8 ± 4.8	−1.6 ± 1.3	35.6	43.6 ± 6.9	−4.0 ± 4.7	−1.0 ± 1.2	15.3

Values are % or mean ± SD.

Abbreviations: HAD, height-for-age difference; HAZ, height-for-age *z*-score.

1 Or placement in foster care.

2 Four studies—Rutter [28], Palacios et al. [18], Matthews et al. [31], and Canzi et al. [19]—expressed age in months as whole numbers; in these cases, we adjusted age by adding a 0.5 mo [28].

3 Values are reported for observations that were included in the subgroup analyses (i.e., contributing a minimum of 5 children per subgroup). This explains the lower total number of observations.

At baseline mean age was around 15 mo of age, but ages varied widely across studies (Table 2). About a third (36%) of children were adopted after 24 mo of age. Mean child stature was 3.9 cm below the median WHO growth standard at baseline, and around one-third of the study children were stunted. Mean exposure to the adoption or foster care environment was 15.8 mo (range: 4.2–27.3 mo). At follow-up, after this exposure, mean HAD was −2.2 cm, and the prevalence of stunting 10%.

**FIGURE 2:**
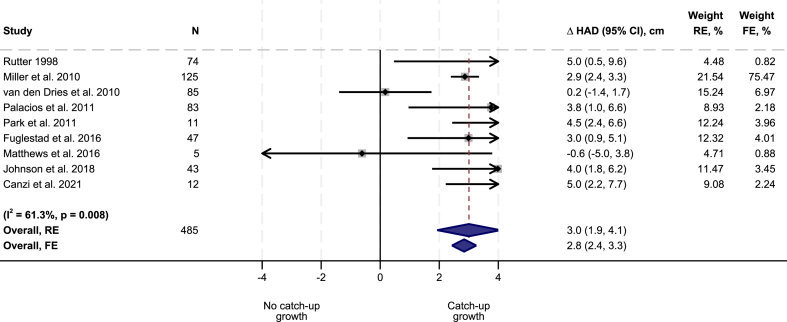
Effect of adoption on height-for-age difference (HAD). RE, random effect; FE, fixed effect.

**FIGURE 3:**
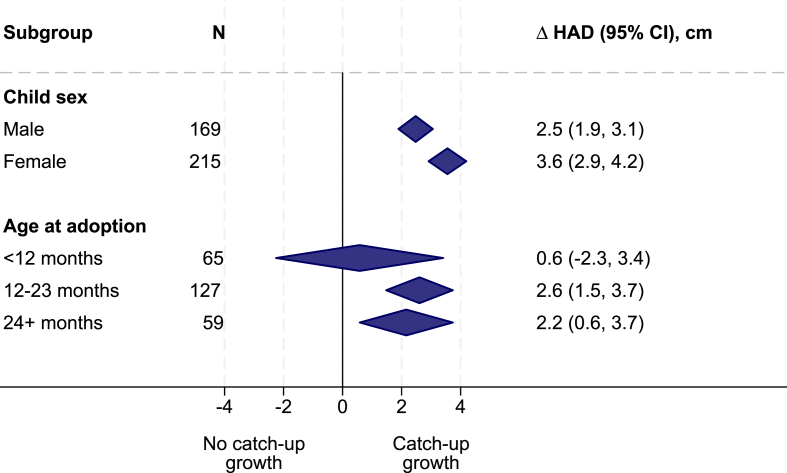
Effect of adoption on height-for-age difference (HAD) by child sex and age at adoption. RE, random effect; FE, fixed effect.

Adoption was positively associated with a change in HAD of 3.0 cm (95% confidence interval [CI]: 1.9, 4.1 cm) adjusting for age and sex (Figure 2). The change in HAD was seen in both boys and girls with a larger point estimate (1.1 cm; *P* = 0.015) in girls (3.6 cm; 95% CI: 2.9, 4.2 cm) than in boys (2.5 cm; 95% CI: 1.9, 3.1 cm) (Figure 3). The point estimates were larger in children adopted after the age of 12 mo (2.6 cm; 95% CI: 1.5, 3.7 cm in children 12–23.9 mo; 2.2 cm; 95% CI: 0.6, 3.7 cm in children above 24 mo) than in children adopted before the age of 12 mo (0.6 cm; 95% CI: −2.3, 3.4 cm).

The use of fixed-effects models did not alter any of the substantive findings. Our deviations from the registered protocol did not change any of our conclusions: when restricting observations to children with baseline measurements taken no later than 1 mo after adoption (or foster care placement), we found a change in HAD of 3.2 cm (95% CI: 2.6, 3.8 cm) (Supplemental Figure 1).

Of the 9 studies included in this analysis, 3 were considered to have low risk of bias (Supplemental Figure 2). When limiting the analyses to these studies, the estimated adjusted change in HAD was 2.7 cm (95% CI: −0.5, 5.9 cm), close to the overall point estimates (Supplemental Figure 3).

The intention-to-treat analysis of the BEIP randomized trial found impacts on HAD of 0.9 cm (95% CI: 0.0, 1.7 cm) at 30 mo of age and 3.0 cm (95% CI: 2.1, 3.8 cm) at 42 mo of age (Table 3). The HAZ estimates are provided Supplemental Table 2 and Supplemental Figures 4–7. The *I*^2^ of the main analysis suggests substantial heterogeneity.

**TABLE 3:** Intention-to-treat analysis of the Bucharest Early Intervention Project randomized trial [25].

HAD (cm)	Institutionalized group *n* = 62		Foster care group *n* = 63		Foster care vs. institutionalized groups
	Round-specific mean	Follow-up vs. baseline	Round-specific mean	Follow-up vs. baseline	Follow-up vs. baseline
Baseline	−3.5 (−4.4, −2.6)	Ref.	−3.4 (−4.3, −2.5)	Ref.	Ref.
30-mo visit	−4.2 (−5.0, −3.3)	−0.7 (−1.4, 0.1)	−3.2 (−4.1, −2.4)	0.2 (−0.5, 0.9)	0.9 (0.0, 1.7)∗
42-mo visit	−4.6 (−5.6, −3.7)	−1.1 (−2.2, 0.0)∗	−1.6 (−2.6, −0.6)	1.8 (0.8, 2.9)∗∗	3.0 (2.1, 3.8)∗∗

Estimates are mean (95% CI). Values were estimated using a mixed model with child as a random effect and treatment group, time, interaction of treatment and time, child age, and sex as fixed effects. We used all available observations at baseline and the 2 follow-up periods in both arms, including measurements in the foster care arm that were taken >3 mo before foster care placement. Biologically implausible height values were dropped. ∗*P* < 0.05; ∗∗*P* < 0.01.

Five adoption (*n* = 371) studies reported changes in child development using 3 different development measures (Table 4) [12,25,[27], [28], [29], [30], [31], [32], [33]]. Adoption was associated with a reduction in the cognitive and motor development gap of ∼46% (range: 43%–50%) and 46% (range: 0%–66%), respectively. Catch-up in linear growth in the subset of studies with child-development outcomes was like that across all studies.

**TABLE 4:** Child development outcomes by study.

Parent study and additional studies	*N*^1^	Child age^2^ (mo)	Development measure	Baseline and follow-up	% Reduction in gap by domain			Comments
					Combined	Cognitive	Motor	
Rutter, 1998 [28]	91	BL: 7 FU: 48	Denver Developmental Scales	BL: 63; FU: 107 (UK adoptees: FU: 117.7)	80	—	—	BL values obtained retrospectively through parent recall Gap defined using comparison group mean (117.7)
Miller et al., 2010 [27] Kroupina et al., 2012 [32] Kroupina et al., 2015 [33]	72	Kroupina et al., 2012 [32] BL: 17 FU: 23	Mullen Scales of Early Learning	Visual reception BL: 38 (gap = 12); FU: 44 (gap = 6) Fine motor BL: 42 (gap = 8); FU: 47 (gap = 3)	—	50	63	The 2 studies by Kroupina et al. [32,33] appear to report on the same children
	46	Kroupina et al., 2015 [33] BL: 19 FU: 25	Mullen Scales of Early Learning	Visual reception BL: 36.33 (gap = 13.67); FU: 43.02 (gap = 6.98) Fine motor BL: 39.61 (gap = 10.39); FU: 46.20 (gap = 3.80)	—	49	63	
van den Dries et al., 2010 [29]	92	BL: 15 FU: 19	Dutch Bayley Scales of Infant Development Second Edition	Mental score BL: 78.77 (gap = 21.23); FU: 87.98 (gap = 12.02) Motor score BL: 88.76 (gap = 11.24); FU: 87.46 (gap = 12.54)	—	43	0	Means weighted across children who were institutionalized and in foster care before adoption
Park et al., 2011 [30]	58	BL: 18 FU: 27	Bayley Scales of Infant Development Second Edition	Mental score BL: 78.4 (gap = 21.6); FU: 88.0 (gap = 12.0) Motor score BL: 84.9 (gap = 15.1); FU: 94.9 (gap = 5.1)	—	44	66	—
Fuglestad et al., 2016 [12]	58	BL: 13 FU: 19	Bayley Scales of Infant Development Third Edition	Cognitive score BL: 89 (gap = 11); FU: 94 (gap = 6) Motor score BL: 83 (gap = 17); FU: 92 (gap = 8)	—	45	53	—
Matthews et al., 2016 [31]	—	—	—	—	—	—	—	No studies on development outcomes found
Johnson et al., 2018 [25]	—	—	—	—	—	—	—	None of the studies on child development provide before and after measures which are needed to assess catch-up
Mean^3^					80	46	46	

Abbreviations: BL, baseline; FU, follow-up.

1 Actual samples size for developmental tests not always clearly reported.

2 Actual ages for those children with reported development data not always clearly reported.

3 Mean calculated using one of the studies by Kroupina et al. [32].

## Discussion

Our analysis of individual data from 9 adoption studies provides clear evidence of partial catch-up in linear growth: adoption from a poor to a favorable environment reduced children’s accumulated height deficit by ∼3 cm. Reducing the accumulated height deficit required children to grow considerably faster (i.e., at a higher velocity) than expected for their age and sex. The median expected increase in height between 15.8 and 32.3 mo (i.e., the mean ages at baseline and follow-up) from the WHO growth standard is around 14 cm [13]. Children in the included studies grew at a 22% higher velocity.

Substantial catch-up in linear growth was found for both boys and girls, but the point estimate for girls was 1.1 cm larger than that for boys. The linear growth deficit in girls before adoption was similar in boys and girls (Table 1), which means that the difference was not due to a higher potential to benefit in girls. Boys are more affected by malnutrition than girls [34], but the mechanisms underlying these differences are not well understood. Boys’ vulnerability may have also limited their (biological) ability to respond to the adoption intervention. An alternative explanation could be that adopted girls received better care than adopted boys did or that girls responded more behaviorally to the care. Evidence from several controlled nutrition trials supports the biological explanation. A meta-analysis demonstrated that the effect of multiple micronutrient supplementation during pregnancy on neonatal mortality was limited to girls [35]. In another meta-analyses, small-quantity lipid-based nutrient supplements reduced the prevalence of stunting and wasting more in girls than in boys, even though the prevalence of these types of undernutrition was higher among boys than that among girls in the control group [36].

Catch-up growth was larger in children adopted after 12 mo of age than that in children adopted at younger ages, although the CI for the estimate at younger ages was quite wide. The smaller accumulated linear growth deficit in children under 12 mo of age may have limited the potential to benefit from adoption.

The catch-up growth found in children adopted after the age of 24 mo suggests that the potential to partially recover lost growth is not limited to younger children. A commonly held view in the nutrition community has been that linear growth retardation is largely irreversible after 24 mo of age. The strong focus on the “first 1000 days” (i.e., pregnancy to 24 mo of age) is based on evidence from a trial that demonstrated that interventions beyond this age have little or no impact on linear growth [37], the finding that HAZ declines rapidly up to the age of 24 mo in malnourished populations [38], and data demonstrating that linear growth retardation starts in utero [39]. Recently, however, it has been shown that the drop in HAZ ≤24 mo followed by a plateau is a statistical artifact [14]. Using the correct metric, that is, HAD, growth faltering continues well beyond 24 mo of age, at least to 60 mo of age. The lack of impact of interventions on linear growth after 24 mo of age has been challenged too. A recent systematic review and meta-analysis demonstrated that zinc, vitamin A, and multiple micronutrient supplements and interventions increasing protein intake improved linear growth in children older than 24 mo [40]. Our results further confirm that the biological window of opportunity for growth does not close at 24 mo of age. These results are in line with current understanding of the biology of growth, that is, there are no fundamental changes in the underlying mechanisms around the age of 24 mo [41].

We do not know if the improvement in growth was sustained through adolescence and adulthood. Furthermore, the evidence of substantial catch-up in height does not imply that the adopted children recovered from the other negative consequences of the deficient environment they experienced early in life. It is not clear, for instance, to what extent the accelerated linear growth in adopted children was accompanied by favorable changes in organ size and function. Even though comparing the magnitude of the association across domains is challenging, the available evidence suggests that catch-up in developmental outcomes was smaller than that in linear growth following adoption. Adoption was associated with a reduction in the accumulated development gap of ∼46% for both cognition and motor skills. This was considerably smaller than the 77% reduction (3 cm reduction from 3.9 cm deficit before adoption) in the linear growth deficit.

Longer-term follow-up studies on children from the 2 Romania studies showed permanent negative effects on the brain. Romanian children who were adopted into UK families had smaller total brain volume and altered brain structure at young adulthood compared with institutionalized UK children who were adopted [28,42]. These changes were associated with lower intelligence and attention deficit/hyperactivity disorder symptoms. Romanian children placed in foster care in Romania [24] experienced lasting negative effects on gray and white brain matter and on cognitive function at 8 y of age [43,44]. The deprivation in the institutional setting in Romania before adoption was not limited to poor nutrition and health and inadequate care practices but was characterized by extreme deprivation and neglect [28] greater than what poor children typically experience in low-income and middle-income countries.

Authors from earlier observational studies have concluded that catch-up growth should be promoted as a strategy to improve child development [2,4]. The evidence presented in this study demonstrated that the recovery in cognitive outcomes was smaller than that observed in stature. Second, there is no known biological mechanism that would explain a causal effect of linear growth on cognition [1]. In addition, the impact of available nutrition interventions on linear growth is too small to result in catch-up growth [7]. Finally, improving child development through direct interventions has been demonstrated to be more effective than addressing these outcomes indirectly through nutrition interventions [45]. Ideally, interventions should be designed to provide both the developmental and nutrition inputs children need.

Only 1 randomized study was included in our analyses. Catch-up in stature and development in the observational studies could have been due to factors unrelated to adoption. It is unlikely, however, that an intervention happened at the same time as the adoption that could have had effects of this magnitude. The included studies provided limited information on how adoptees were selected. If adoption agencies had systematically selected children with a high (or low) potential to benefit in growth or development, our estimates would be biased upward (or downward). It is unlikely that selection procedures would have been based on potential to benefit. Furthermore, we are unaware of any markers for this potential to benefit in linear growth. Another potential source of selection bias could be the adopting parents who agreed to participate in the study, but it is difficult to determine to what extent that may have biased the results. Parents with better caregiving and nurturing skills might have been more inclined to participate which could have biased our estimates upward. It is also possible, however, that parents of children requiring more medical or other care were more prone to participate, which would have led to a downward bias. Finally, several studies did not follow standard procedures for anthropometric assessments (such as standardizing enumerators and measuring recumbent length in children under 2 y and standing height in older children) [46], which could have introduced noise but is unlikely to have biased the impact estimates. Notwithstanding the limitations, we are confident that our findings are not simply a consequence of confounding or bias. Limiting the analyses to the 3 studies with the lowest risk of bias did not alter the linear growth findings. In addition, the findings are in line with our current understanding of linear growth: greater changes were found in the age groups with the largest potential to benefit. In addition, larger point estimates were found in girls than those in boys, which is in line with emerging findings on this topic [35,36]. Finally, the findings from the included RCT [25], which did not have many of the above-listed limitations, are like those from the meta-analysis.

Our analyses demonstrate that catch-up in linear growth in children under the age of 5 y is biologically possible when the environment children are exposed to is improved profoundly and comprehensively. Nutrition interventions as currently implemented do not provide the intensity, duration, and breadth needed for catch-up growth to occur. It is reasonable to assume that the lives of children after adoption improved across a wide range of domains, including health, nutrition, responsive caregiving, opportunities for learning, and safety and security, that is, all elements of a nurturing care environment necessary for children to reach their full development potential [47]. Our findings suggest, however, that the partial reversal of the accumulated height deficit was larger than the recovery in developmental outcomes. Notwithstanding the demonstrated potential for partial reversal of deficits in linear growth, scientific, program, and policy efforts should focus on ensuring that all children grow and develop in fully supportive environments that prevent deficits from occurring rather than trying to correct deficits and consequences that have occurred because of deficient environments.

## Author contributions

The authors’ responsibilities were as follows – JLL: conceptualized the study; JLL, MDA: designed the study and drafted the text; MDA: conducted the data analyses; EAF: revised it critically for content; and all authors: critically reviewed and interpreted the analytic findings and read, edited, and approved the final manuscript. JLL is responsible for the overall content as guarantor. The corresponding author (JLL) attests that all listed authors meet authorship criteria and that no others meeting the criteria have been omitted.

## Data availability

Data described in the article will not be made available because they are compiled from 9 different studies and access is under the control of the investigators of each of those studies.

## Funding

The study received financial support from the CGIAR Research Program on Agriculture for Nutrition and Health (A4NH), led by IFPRI. The funders of the study had no role in study design, data collection, data analysis, data interpretation, or writing of the report.

## Conflicts of interest

JLL reports financial support was provided by Bill & Melinda Gates Foundation and by CGIAR Research Program on Agriculture for Nutrition and Health (A4NH), led by IFPRI. All other authors report no conflicts of interest.
