# Supplementary material for: Adoption or Placement in Foster Care and Catch-up in Linear Growth and Development: A Meta-Analysis of Individual Participant Data
Source: Adv Nutr. 2025 Feb 22;16(4):100395. doi: 10.1016/j.advnut.2025.100395 (PMC11957773; doi:10.1016/j.advnut.2025.100395)
Supplement: multimedia component 1 [file mmc1.docx]

**Adoption or placement in foster care and catch-up in linear growth and development: a meta-analysis of individual participant data**

Jef L. Leroy et al.

**Supplemental online material**

**Supplemental Table S1:** Studies included in the 2020 literature review on catch-up growth (10) and studies included in the present meta-analysis.

| **Study** | **Included in 2020 literature review** | **Included in present meta-analysis** | **Reasons why not included in meta-analysis** |
| --- | --- | --- | --- |
| Melsen et al. 1986 (11) | Yes | No | Data not available |
| Jenista and Chapman 1987 (12) | Yes | No | No response |
| Proos et al. 1992  (13) | Yes | No | Data not available |
| Benoit et al. 1996 (14) | No | No | Data not available |
| Oostdijk et al. 1996 (15) | Yes | No | Data not available |
| Rutter et al. 1998 (1) | Yes | Yes |  |
| Miller et al. 2010 (2) | Yes | Yes |  |
| van den Dries et al. 2010 (3) | Yes | Yes |  |
| Palacios et al. 2011 (4) | No | Yes |  |
| Park et al. 2011 (5) | No | Yes |  |
| Esposito et al. 2016 (16) | Yes | No | No response |
| Fuglestad et al. 2016 (6) | Yes | Yes |  |
| Matthews et al. 2016 (7) | No | Yes |  |
| Ferrara et al. 2018 (17) | Yes | No | No response |
| Johnson et al. 2018 (8) | Yes | Yes |  |
| Canzi et al. 2021 (9) | No | Yes |  |

**Supplemental Table S2:** Intention-to-treat analysis of the Bucharest Early Intervention Project randomized trial – impact on height-for-age z-score (HAZ)

|  | **Institutionalized group**  **N=62** | | **Foster care group**  **N=63** | | **Foster care vs. institutionalized groups** |
| --- | --- | --- | --- | --- | --- |
|  | **Round-specific mean** | **Follow-up vs. baseline** | **Round-specific mean** | **Follow-up vs. baseline** | **Follow-up vs. baseline** |
| **HAZ** |  |  |  |  |  |
| Baseline | -0.9 (-1.2, -0.7) | ref. | -0.9 (-1.1, -0.6) | ref. | ref. |
| 30-month visit | -1.0 (-1.3, -0.8) | -0.1 (-0.3, 0.1) | -0.7 (-1.0, -0.5) | 0.1 (-0.1, 0.4) | 0.2 (0.0, 0.5) |
| 42-month visit | -1.2 (-1.5, -0.9) | -0.3 (-0.6, 0.0) | -0.4 (-0.7, -0.1) | 0.5 (0.2, 0.8)** | 0.7 (0.5, 1.0)** |

Estimates are mean (95% CI). Values were estimated using a mixed model with child as a random effect, and treatment group, time, interaction of treatment and time, child age and sex as fixed effects. We used all available observations at baseline and the two follow-up periods in both arms, including measurements in the foster care arm that were taken more than three months prior to foster care placement. Biologically implausible height values were dropped. * p value <0.05; ** p value < 0.01

**Supplemental Figure S1:** Effect of adoption on height-for-age difference (HAD) when restricting observations to children with baseline measurements taken no later than one month after adoption (or foster care placement). RE: random effects; FE: fixed effects.
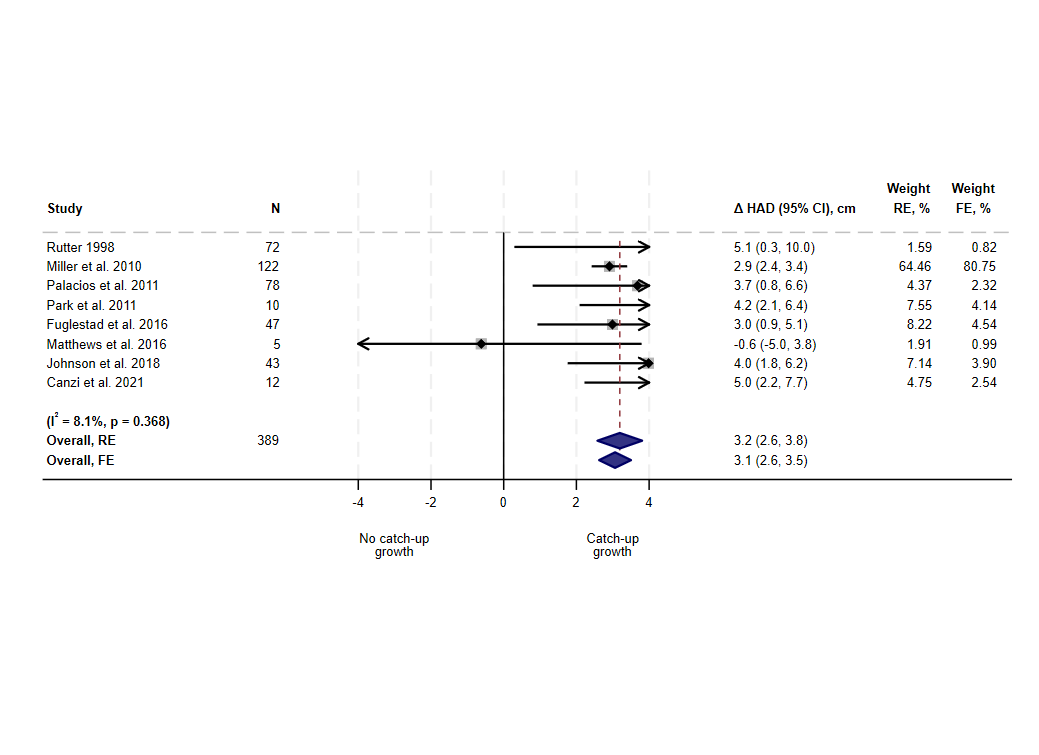


**Supplemental Figure S2**: Risk of bias assessment

**Supplemental Figure S3**: Effect of adoption on height-for-age difference (HAD) in 3 low-risk studies. RE: random effects; FE: fixed effects.


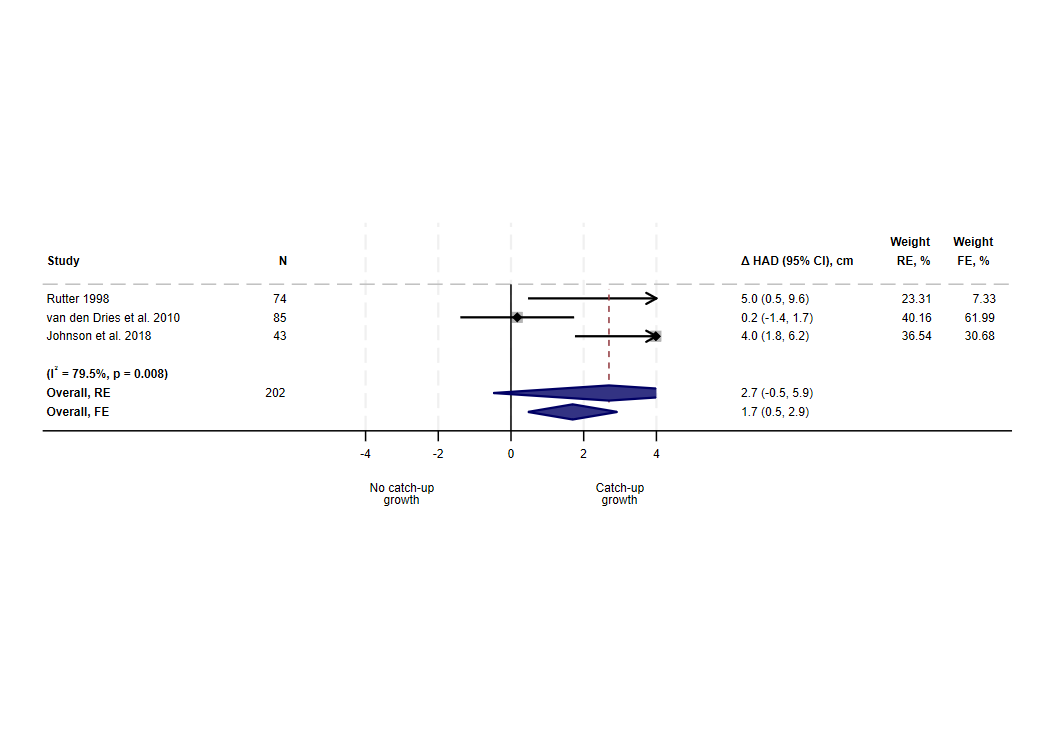


**Supplemental Figure S4**: Effect of adoption on height-for-age z-score (HAZ). RE: random effects; FE: fixed effects.


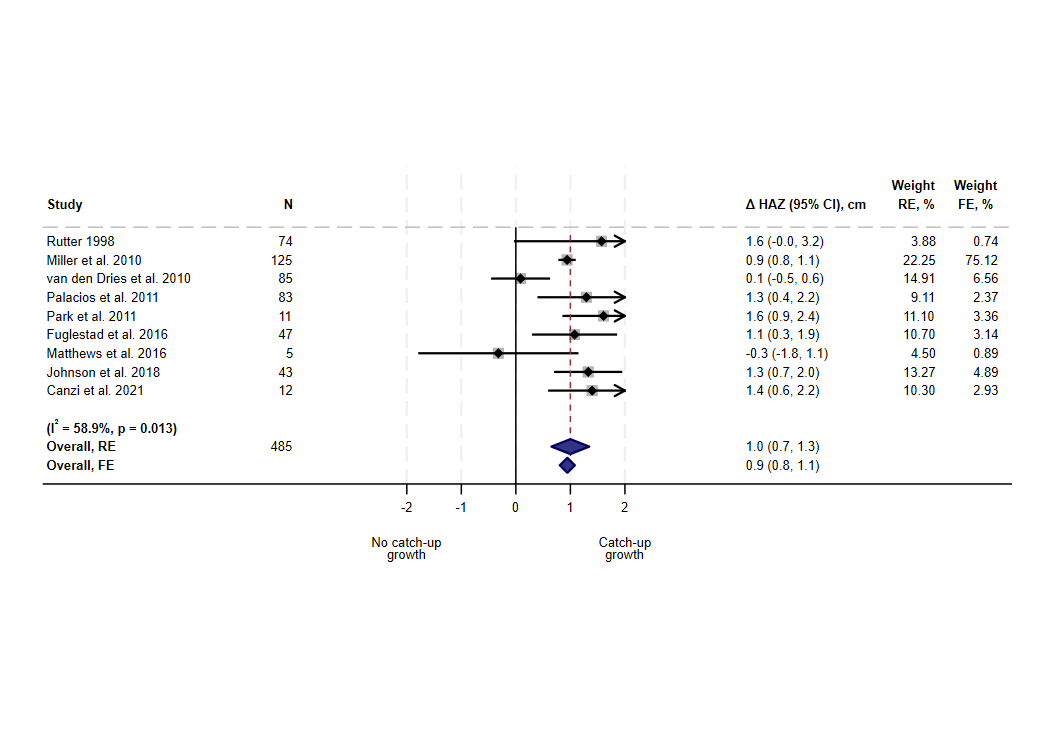


**Supplemental Figure S5**: Effect of adoption on height-for-age z-score (HAZ) by child sex and age at adoption. RE: random effects; FE: fixed effects.

**
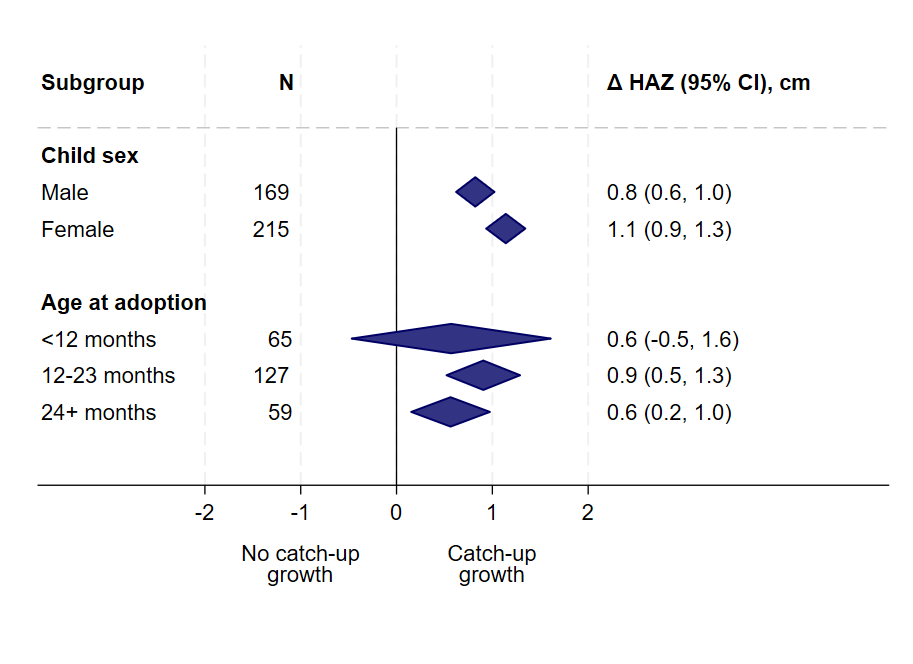
**

**Supplemental Figure S6:** Effect of adoption on height-for-age z-score (HAZ) when restricting observations to children with baseline measurements taken no later than one month after adoption (or foster care placement). RE: random effects; FE: fixed effects.
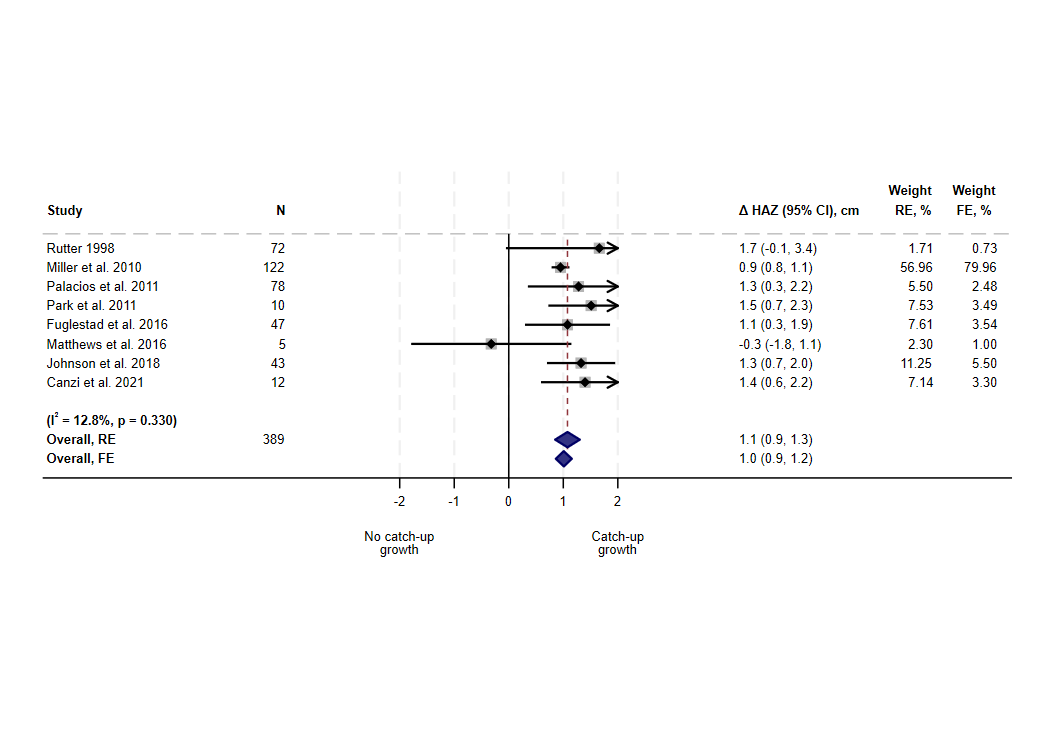


**Supplemental Figure S7**: Effect of adoption on height-for-age z-score (HAZ) in 3 low-risk studies. RE: random effects; FE: fixed effects.


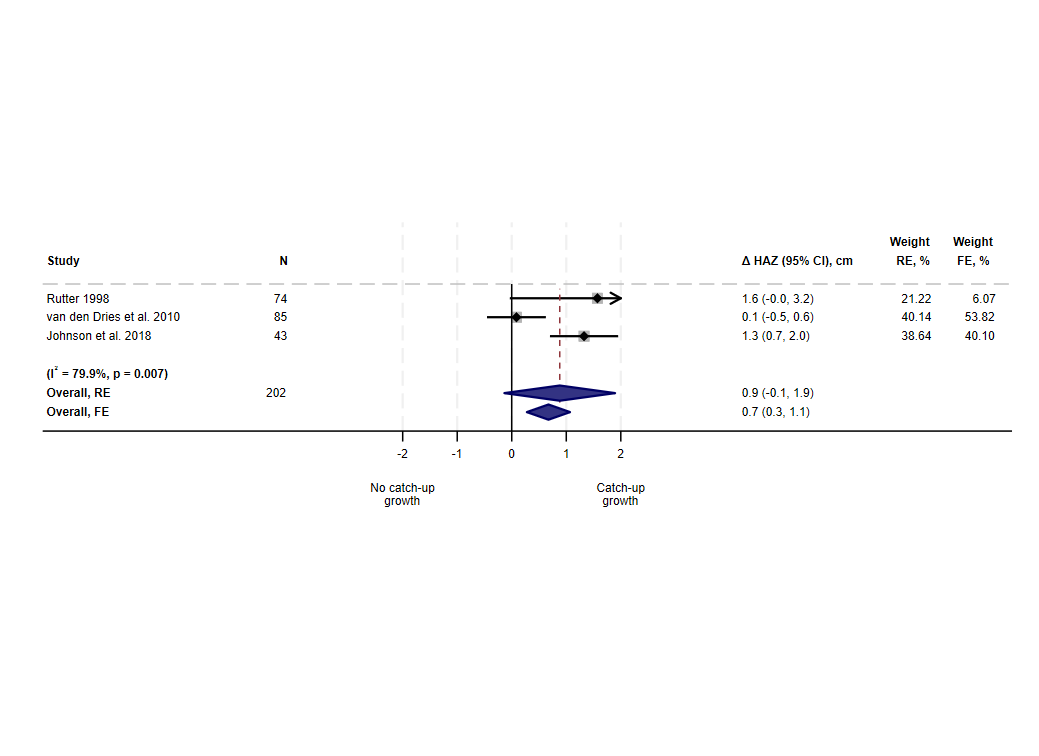


**References**

1. Leroy JL, Frongillo EA, Dewan P, Black MM, Waterland RA. Can children catch up from the consequences of undernourishment? Evidence from child linear growth, developmental epigenetics, and brain and neurocognitive development. Advances in Nutrition. 2020;in press:1032–41.

2. Melsen B, Wenzel A, Miletic T, Andreasen J, Vagn-Hansen PL, Terp S. Dental and skeletal maturity in adoptive children: assessments at arrival and after one year in the admitting country. Ann Hum Biol. 1986;13:153–9.

3. Jenista JA, Chapman D. Medical Problems of Foreign-Born Adopted Children. American Journal of Diseases of Children. 1987;141:298–302.

4. Proos LA, Hofvander Y, Wennqvist K, Tuvemo T. A Longitudinal Study On Anthropometric And Clinical Development Of Indian Children Adopted In Swedenanthropometric And Clinical Development Of Indian Children Adopted In Sweden. Ups J Med Sci. 1992;97:93–106.

5. Benoit TC. Romanian Adoption. Arch Pediatr Adolesc Med. 1996;150:1278.

6. Oostdijk W, Yapm Y, Rekers-Mombarg, LTM Massa G, Brand R, Drop S. The impact of early puberty on final height in foreign born, adopted children in the Netherlands. Central Precocious Puberty and gonadotropin releasing hormone agonist treatment. Erasmus University Rotterdam; 1996.

7. Rutter M, Andersen-Wood L, Beckett C, Bredenkamp D, Castle J, Dunn J, Ehrich K, Groothues C, Harborne A, Hay D, et al. Developmental catch-up, and deficit, following adoption after severe global early privation. J Child Psychol Psychiatry. 1998;39:465–76.

8. Miller BS, Kroupina MG, Mason P, Iverson SL, Narad C, Himes JH, Johnson DE, Petryk A. Determinants of Catch-Up Growth in International Adoptees from Eastern Europe. Int J Pediatr Endocrinol. 2010;2010:1–8.

9. Van Den Dries L, Juffer F, Van Ijzendoorn MH, Bakermans-Kranenburg MJ. Infants’ physical and cognitive development after international adoption from foster care or institutions in China. Journal of Developmental and Behavioral Pediatrics. 2010;31:144–50.

10. Palacios J, Román M, Camacho C. Growth and development in internationally adopted children: extent and timing of recovery after early adversity. Child Care Health Dev. 2011;37:282–8.

11. Park H, Bothe D, Holsinger E, Kirchner HL, Olness K, Mandalakas A. The Impact of Nutritional Status and Longitudinal Recovery of Motor and Cognitive Milestones in Internationally Adopted Children. Int J Environ Res Public Health. 2011;8:105–16.

12. Esposito EA, Koss KJ, Donzella B, Gunnar MR. Early deprivation and autonomic nervous system functioning in post-institutionalized children. Dev Psychobiol. 2016;58:328–40.

13. Fuglestad AJ, Kroupina MG, Johnson DE, Georgieff MK. Micronutrient status and neurodevelopment in internationally adopted children. Acta Paediatrica, International Journal of Paediatrics. 2016;105:e67–76.

14. Matthews JAK, Tirella LG, Germann ES, Miller LC. International adoptees as teens and young adults: family and child function. Early Child Dev Care. 2016;186:1453–65.

15. Ferrara P, Cutrona C, Guadagno C, Amodeo ME, Del-Vescovo E, Ianniello F, Petitti T. Changes in trajectories of physical growth in a domestic adoptees sample: A preliminary study. Turkish Journal of Pediatrics. 2018;60:464–6.

16. Johnson DE, Tang A, Almas AN, Degnan KA, McLaughlin KA, Nelson CA, Fox NA, Zeanah CH, Drury SS. Caregiving Disruptions Affect Growth and Pubertal Development in Early Adolescence in Institutionalized and Fostered Romanian Children: A Randomized Clinical Trial. Journal of Pediatrics. 2018;203:345-353.e3.

17. Canzi E, Rosnati R, Miller LC. Growth recovery in newly arrived international adoptees in Italy: relation to parenting stress. Minerva pediatrics [Internet]. 2021;73:316–23. Available from: http://www.ncbi.nlm.nih.gov/pubmed/29651833
